# Transcriptional Profiling and Functional Analysis of N1/N2 Neutrophils Reveal an Immunomodulatory Effect of S100A9-Blockade on the Pro-Inflammatory N1 Subpopulation

**DOI:** 10.3389/fimmu.2021.708770

**Published:** 2021-08-10

**Authors:** Andreea C. Mihaila, Letitia Ciortan, Razvan D. Macarie, Mihaela Vadana, Sergiu Cecoltan, Mihai Bogdan Preda, Ariana Hudita, Ana-Maria Gan, Gabriel Jakobsson, Monica M. Tucureanu, Elena Barbu, Serban Balanescu, Maya Simionescu, Alexandru Schiopu, Elena Butoi

**Affiliations:** ^1^Biopathology and Therapy of Inflammation, Institute of Cellular Biology and Pathology “Nicolae Simionescu”, Bucharest, Romania; ^2^Department of Clinical Sciences Malmö, Lund University, Malmö, Sweden; ^3^Departament of Cardiology, Elias Emergency Hospital, Carol Davila University of Medicine and Pharmacy, Bucharest, Romania; ^4^Department of Pathophysiology, University of Medicine, Pharmacy, Sciences and Technology of Targu-Mures, Targu-Mures, Romania

**Keywords:** neutrophil polarization, N1 neutrophils, N2 neutrophils, S100A8/A9, ABR-238901, RNA-Seq, neutrophil chemotaxis

## Abstract

Neutrophils have been classically viewed as a homogenous population. Recently, neutrophils were phenotypically classified into pro-inflammatory N1 and anti-inflammatory N2 sub-populations, but the functional differences between the two subtypes are not completely understood. We aimed to investigate the phenotypic and functional differences between N1 and N2 neutrophils, and to identify the potential contribution of the S100A9 alarmin in neutrophil polarization. We describe distinct transcriptomic profiles and functional differences between N1 and N2 neutrophils. Compared to N2, the N1 neutrophils exhibited: i) higher levels of ROS and oxidative burst, ii) increased activity of MPO and MMP-9, and iii) enhanced chemotactic response. N1 neutrophils were also characterized by elevated expression of NADPH oxidase subunits, as well as activation of the signaling molecules ERK and the p65 subunit of NF-kB. Moreover, we found that the S100A9 alarmin promotes the chemotactic and enzymatic activity of N1 neutrophils. S100A9 inhibition with a specific small-molecule blocker, reduced CCL2, CCL3 and CCL5 chemokine expression and decreased MPO and MMP-9 activity, by interfering with the NF-kB signaling pathway. Together, these findings reveal that N1 neutrophils are pro-inflammatory effectors of the innate immune response. Pharmacological blockade of S100A9 dampens the function of the pro-inflammatory N1 phenotype, promoting the alarmin as a novel target for therapeutic intervention in inflammatory diseases.

## Introduction

Neutrophils are the first responders in host defense, with an important role in promoting the innate immune response. They originate from the bone marrow and are released in the circulation when they mature and are stimulated by invasive pathogens and inflammatory signals that facilitate their migration to sites of infection or tissue injury. At the site of infection, neutrophils eliminate the invading pathogens utilizing a combination of NADPH oxidase-derived reactive oxygen species (ROS), cytotoxic granule components, and neutrophil extracellular traps (NETs) (1).

Although regarded for a long time as a homogenous population with conserved phenotype and function, recent evidence has suggested the existence of neutrophil heterogeneity with different functional phenotypes, both in healthy individuals and in pathological conditions including cancer, infections, and autoimmune and inflammatory disorders (2, 3). The heterogeneity of neutrophil populations is characterized by differences in life span, cytokine release, surface proteins, antibacterial responses, as well as pro-inflammatory, proangiogenic, or immunosuppressive functions (2–5). It has been reported that a unique neutrophil population emerging during acute inflammation suppresses T cell function, a process dependent of neutrophil Mac-1 and ROS (6). Infection with *Staphylococcus aureus* leads to two subsets of murine polymorphonuclear neutrophils with important differences in their expression of surface markers, cytokine production and macrophage activation potential (7). In systemic lupus erythematosus and other autoimmune diseases, a subpopulation of low-density neutrophils (LDN) with an unclear physiological role has been detected (8). The LDN population with immunosuppressive properties has also been found to accumulate in tumor-bearing mice and cancer patients. In contrast, the high-density neutrophils (HDN) have been shown to have anti-tumorigenic functions (9). Moreover, circulating neutrophil subsets in advanced lung cancer patients have unique immune signatures and are associated with the disease prognosis (10).

Recently, the consecutive myocardial infiltration of two neutrophil subpopulations has been described in a mouse model of myocardial infarction (MI). Cardiac N1 neutrophils isolated on day one post-MI, during the inflammatory phase, showed high levels of pro-inflammatory markers (CCL3, IL-1β, IL-12a, and TNF-α). In contrast, cardiac N2 neutrophils isolated at days 5 and 7, during the reparatory phase, exhibited increased expression of anti-inflammatory markers CD206 and IL-10. Moreover, neutrophils polarized *in vitro* with a combination of lipopolysaccharide (LPS) and interferon-*γ* (IFN-*γ*) for N1 or interleukin-4 (IL-4) for N2, exhibited similar markers as the sub-populations found *in vivo* (11). Uncovering the potentially important role of the different neutrophil subtypes in driving inflammation or the resolution of inflammation could have significant therapeutic relevance, as targeting a specific subpopulation may modulate the course of the disease.

S100A8/A9 is an immune mediator abundantly secreted by neutrophils that plays a complex role in various pathologies with an immune and inflammatory component. S100A9 and its dimerization partner S100A8 are rapidly released as the S100A8/A9 heterodimer upon cell activation (12) and functions as a damage-associated molecular pattern (DAMPs) that binds to toll-like receptor 4 (TLR4) (13), and to the receptor for advanced glycation end products (RAGE) (14). Activation of TLR4 by S100A8/A9 has been shown to have an important pro-inflammatory role in the pathogenesis of endotoxin-induced shock (15), autoimmune disease and cancer (16).

After MI, S100A8/A9 is abundantly secreted by activated neutrophils and promotes cardiac inflammation by stimulating myeloid cell production and trafficking to the ischemic myocardium (17). We have recently found that short-term S100A9 blockade with the specific blocker ABR-238901 during the inflammatory phase of MI reduces myocardial and systemic inflammation, and improves cardiac function (17). The precise mechanisms behind these beneficial therapeutic effects remain to be investigated. Interestingly, binding of S100A8/A9 to TLR4 on neutrophils has subsequently been shown to drive IL-1β production, leading to increased myelopoesis in MI (18). As IL-1β secretion is characteristic for the N1 neutrophil phenotype, we hypothesize that S100A8/A9 might play an important role in the development of this particular subpopulation.

In this work, our main aims were: i) to perform a comparative study of N1 and N2 neutrophil genotype, phenotype and function, and ii) to investigate the effects of S100A9 blockade with ABR-238901 on the functions of the two neutrophil subpopulations. Elucidating the immunomodulatory properties of S100A9 inhibition is highly relevant for further development of the compound toward a potential anti-inflammatory treatment in MI and other immune and inflammatory diseases.

## Materials and Methods

### Mice

Male and female C57BL/6J mice, between 12-16 weeks old, were bred and housed in pathogen-free conditions at the Institute of Cellular Biology and Pathology (ICBP) “Nicolae Simionescu”. The mice were euthanized through cervical dislocation, and the femurs and tibias were collected in a Petri dish containing ice-cold RPMI 1640 supplemented with 10% FBS and 1% Penicillin/streptomycin, for further isolation of bone marrow.

All animal experiments were performed in strict accordance with the European Guidelines for animal welfare (Directive 2010/63/EU) and approved by The National Sanitary Veterinary and Food Safety Authority (nr. 425/22.10.2018). All procedures were approved by the Institutional Ethics Committee of ICBP “N. Simionescu” (Bucharest, Romania).

### Isolation and Polarization of Neutrophils

#### Isolation of Neutrophils

Cells were isolated from mouse bone marrow by Percoll gradient centrifugation, using a simplified and improved version of a previous protocol (19). Briefly, the bones were placed in HBSS-Prep to prevent drying, the ends were cut and the bone marrow (BM) was flushed into a 50 ml conical tube with HBSS-Prep and centrifuged at 400 × g for 5 min. For erythrocyte lysis, the pellet was resuspended in 10 ml NaCl 0.2% for 30-40 s and the osmolarity was then restored with 10 ml 1.6% NaCl. The resulting suspension was centrifuged in 62.5% Percoll in HBSS-Prep for 30 min at 1000 × g, without brake. At the end of centrifugation, the neutrophils-containing pellet was transferred to another 15 ml tube, washed twice with HBSS and cells were resuspended in RPMI. The purity of isolated neutrophils was confirmed by flow cytometry using the neutrophil marker Ly-6G and by fluorescence microscopy using Hoechst/PI staining (**Figure S1**).

#### Polarization of Neutrophils

Freshly isolated neutrophils were pooled from 6–10 mice and cultured for 2h/18h in RPMI medium, in the presence of 100 ng/ml lipopolysaccharide (LPS) and 20 ng/ml interferon gamma (IFNγ) or 20 ng/ml interleukin 4 (IL-4) - in order to obtain polarized N1 (inflammatory) and N2 (anti-inflammatory) neutrophil subsets respectively. This polarization protocol has previously been shown by Ma et al. to generate neutrophil sub-populations with a similar phenotype as the N1/N2 neutrophils isolated from infarcted hearts *in vivo* (11). Unstimulated neutrophils were used as controls (N). In the experiments when S100Ab was blocked, N1 and N2 neutrophils were polarized in the presence of the S100A9 inhibitor ABR-238901 (100µM, Active Biotech AB, Sweden).

### mRNA-Sequencing

To profile the gene expression of N1 and N2 neutrophils after 2h polarization, we used 3 samples per condition and 20x10^6^ neutrophils per sample, pooled from several mice. Total RNA was isolated using TRIzol reagent and Phasemaker Tubes (Thermo Fischer, Waltham, Massachusetts, US) and was sent to Novogene (Cambridge, UK) for mRNA-seq analysis. RNA degradation and contamination were monitored on 1% agarose gels. RNA purity was checked using the NanoPhotometer^®^ spectrophotometer (IMPLEN, CA, USA). RNA integrity and quantitation were assessed using the RNA Nano 6000 Assay Kit with the Bioanalyzer 2100 system (Agilent Technologies, CA, USA). A sample from the LPS+IFNγ polarization did not pass the quality control test and was excluded from the downstream analysis.

A total amount of 1 µg RNA per sample was used as input material for RNA analysis. Sequencing libraries were generated using the NEBNext UltraTM RNA Library Prep Kit for Illumina (NEB, USA) following the manufacturer’s recommendations and index codes were added to attribute sequences to each sample. Library quality was assessed on the Agilent Bioanalyzer 2100 system (Agilent Technologies, CA, USA). The clustering of index-coded samples was performed on a cBot Cluster Generation System using the PE Cluster Kit cBot-HS (Illumina). After cluster generation, the library preparations were sequenced using Illumina NovaSeq 6000 (Illumina) and paired-end reads were generated.

Raw data (raw reads) of FASTQ format were first processed through fastp (20). Clean data were obtained by removing reads containing adapter and poly-N sequences and reads with low quality from raw data. Simultaneously, Q20, Q30 and GC content of the clean data were calculated (**Supplementary Table 1**). Paired-end clean reads were aligned to the Ensembl mouse reference genome (GRCm38.p6) (21) using the Spliced Transcripts Alignment to a Reference (STAR) software (22). A summary of the mapping result is presented in (**Supplementary Table 2**). Gene expression values FPKM (expected number of Fragments Per Kilobase of transcript sequence per Millions base pairs sequenced) were calculated and used for the PCA and Pearson correlation coefficient matrix, using R software (23).

### Differential Expression Analysis

Differential expression analysis was performed using the DESeq2R package (2_1.6.3) (24). The resulting P values were adjusted using Benjamini and Hochberg’s approach for controlling the false discovery rate (FDR). Genes with an adjusted P-value <0.05 found by DESeq2 were assigned as differentially expressed (DEGs). Using a built-in R package, pheatmap, a hierarchical clustering heatmap was generated presenting the log2(FPKM+1) of DEG union within all comparison groups. Volcano plots were realized using EnhancedVolcano R package (25).

### Functional Analysis of DEGs

Functional enrichment analysis of the up-regulated N1 gene cluster was performed using g:GOSt function in gProfiler version e102_eg49_p15_7a9b4d6, database updated on 15/12/2020 (26). The selected organism was *Mus musculus*, the significance threshold was g:SCS, with a user threshold of 0.01. Gene Ontology, pathways from KEGG, Reactome and regulatory motif matches from TRANSFAC databases were inquired.

GO enrichment and KEGG database enrichment analysis was performed using the clusterProfiler R package (27) on all the DEGs, either down or up-regulated and the terms with a corrected P value less than 0.05 were considered significantly enriched.

### Quantitative RT-PCR

Validation of key molecules found to be highly increased by RNA-seq was performed by qPCR using RNA obtained from pooled neutrophils isolated from subsequent experiments. Total cellular RNA was extracted from N, N1 and N2 neutrophils using TRIzol or Qiagen PureLink RNA Kit (Ambion™, Carlsbad, CA). First-strand cDNA synthesis was performed employing 1 μg of total RNA and MMLV reverse transcriptase, according to the manufacturer’s protocol (Invitrogen). Assessment of mRNA expression was done by amplification of cDNA using a LightCycler 480 Real-Time PCR System (Roche) and SYBR Green I. The primer sequences for the mRNAs of interest are shown in **Supplementary Table 3**. The relative quantification was done by the comparative CT method and expressed as arbitrary units. Beta-actin was used as reporter gene.

### Cytokine Array

The presence of soluble pro-inflammatory cytokines and chemokines in the neutrophil condition media was analyzed using the Proteome Profiler Mouse Cytokine Array Kit (ARY006, R&D Systems) in conditioned media from the N, N1 and N2 subpopulations. Detection of the chemiluminescent signal was performed using the Luminescent image analyzer LAS 4000 (Fujifilm). The mean pixel density of each point was calculated using ImageJ (Bethesda, MD).

### Enzyme Linked Immunosorbent Assay (ELISA)

The supernatant was harvested from control (N) or polarized N1 and N2 neutrophils cultured in the presence or absence of ABR-238901 (100 µM). We measured the amount of the proteins of interest released in the condition media by using specific kits (R&D Systems & Mabtech), following the manufacturer’s instructions.

### Measurement of Reactive Oxygen Species

Control (N) or activated neutrophils (N1 and N2) were assayed for intracellular ROS using 2′,7′-dichlorofluorescein diacetate (DCFH-DA) as previously described (28). Briefly, the cells were incubated with 5 μM DCFH-DA (30 min at 37°C) and the DCF fluorescence emission was detected at 535 nm with an excitation wavelength of 485 nm in a 96-well microplate reader (GENios, Tecan). Immediately after DCF measurements, cells were further incubated for 20 min with Hoechst 33342 (0.2 µg/ml) and the fluorescence was measured at 460 nm (with an excitation wavelength of 345 nm). ROS was expressed as DCF/Hoechst fluorescence units.

### The Cellular Energetics of N1 and N2 Neutrophils

An XFp Extracellular Flux Analyzer (Seahorse, Agilent Technologies) was used to measure the oxygen consumption rate (OCR) and the proton efflux rate (PER) as a measure of extracellular acidification in control (N) or polarized neutrophils (N1 and N2). Immediately following isolation, neutrophils were added at 5 x 10^5^ cells/well onto a poly-L-lysine coated XFp plate and stimulated for 2 h to obtain polarized N1 and N2 neutrophil subsets. The OCR and PER were measured in XF media (non-buffered DMEM containing 10 mM glucose, 4 mM L-glutamine, and 2 mM sodium pyruvate) under basal conditions and in response to phorbol 12-myristate 13-acetate (PMA) activation. After 3 h, 500 nM Rotenone was injected to measure the amount of OCR due to mitochondrial activity. After the measurements, data were normalized to the cell number by staining the cells for 10 min with Hoechst 33342 (5 µg/ml) followed by measurement on a microplate reader (GENios, Tecan). The assay was performed twice in duplicates for each condition.

### Determination of Cell Migration by Chemotaxis Assay

Real-time migration was monitored using CIM-plate-16 and the xCELLigence System RTCA DP Instrument (Roche). We used a 16-well modified Boyden chamber composed of an upper chamber (UC) and a lower chamber (LC) that snapped together to form a tight seal. The bottom of the UC consists of a microporous polyethylene terephthalate membrane that permits the translocation of cells from the upper to the bottom side. Cell migration was monitored by interdigitated gold microelectrode sensors that generate an impedance signal by contact with the migrated cells. IL-8 (300ng/ml) was added as a chemoattractant in the LC. We seeded 4x10^5^ neutrophils in the UC of the CIM-plate-16 in RPMI medium without FCS. Cell migration was monitored for up to 20 h.

### Gelatin Zymography

The gelatinolytic activity of the MMP‐9 released by N, N1 and N2 neutrophils in the culture medium was evaluated by gelatin zymography, as previously described (29). Briefly, the cell culture medium was collected and the nonreducing Laemmli’s buffer was added to the cell‐free neutrophil supernatants and subjected to electrophoresis under non‐reducing conditions on 10% polyacrylamide gels containing 1 mg/mL gelatin as substrate. After electrophoresis, the gels were re‐natured in 2.5% Triton X‐100 (2 × 30 minutes) and incubated with 50 mmol/L Tris‐HCl pH 7.4, containing 10 mmol/L CaCl_2_ and 0.2 mmol/L PMSF (18 hours, 37°C). The gels were subsequently stained with 0.2% Coomassie brilliant blue R‐250 and de‐stained with 10% acetic acid and 25% methanol. The white bands against the blue background were indicative of the gelatinolytic activity of MMP-2/-9. Image acquisition was done with a transillumination imaging system LAS 4000 (Fujifilm). Data are presented as fold increase over the unstimulated control.

### Western Blot

Following polarization, neutrophils were rapidly chilled by the addition of ice‐cold HBSS. Neutrophils were pelleted, supernatants were collected and the cell pellets were lysed using RIPA lysis buffer supplemented with a protease inhibitor cocktail. After centrifugation (12000 × g), the proteins were quantified by bicinchoninic acid (BCA) Protein Assay Kit. Samples (30 μg protein) were separated on 10% SDS-PAGE (sodium dodecyl sulfate-polyacrylamide) gel electrophoresis and transferred to nitrocellulose membranes, which were subsequently probed with specific antibodies. The signals were visualized using SuperSignal West Pico chemiluminescent substrate (Pierce) and quantified by densitometry employing the gel analyzer system Luminescent image analyzer LAS 4000 (Fujifilm) and the Image reader LAS 4000 software.

### Detection of Myeloperoxidase (MPO) and Nitric Oxide (NO)

Quantification of MPO activity was performed in the cell lysate, and of NO levels in the conditioned media, using specific kits from Elabscience (K074 and K035, respectively). Following polarization, conditioned media from N, N1, N2 neutrophils were harvested and used for NO detection, while neutrophils were rapidly chilled with ice‐cold PBS, centrifugated and the resulted cell lysate used for MPO quantification according to the manufacturer’s instructions.

### Statistical Analyses

GraphPad Prism 7.0 with data points expressed as mean ± standard deviation (SD) was used for all statistical analyses. We used a two-tailed Student’s t-test when comparing two experimental groups and a one-way ANOVA and Tukey’s multiple comparison test when comparing more than two groups. A p-value of p<0.05 was considered statistically significant.

### Data Access

All sequencing data have been deposited in the ArrayExpress database, https://www.ebi.ac.uk/arrayexpress/E-MTAB-10508. All other data are available from the authors on request.

## Results

### N1 and N2 Neutrophils Exhibit a Distinct Transcriptomic Profile

RNA obtained from freshly isolated neutrophils polarized for 2h with LPS+IFNγ (N1) or IL-4 (N2), was analyzed by RNA-seq. The sample replicates showed a high degree of correlation, as determined by Pearson correlation matrix (**Figure S1**).

**Figure 1:**
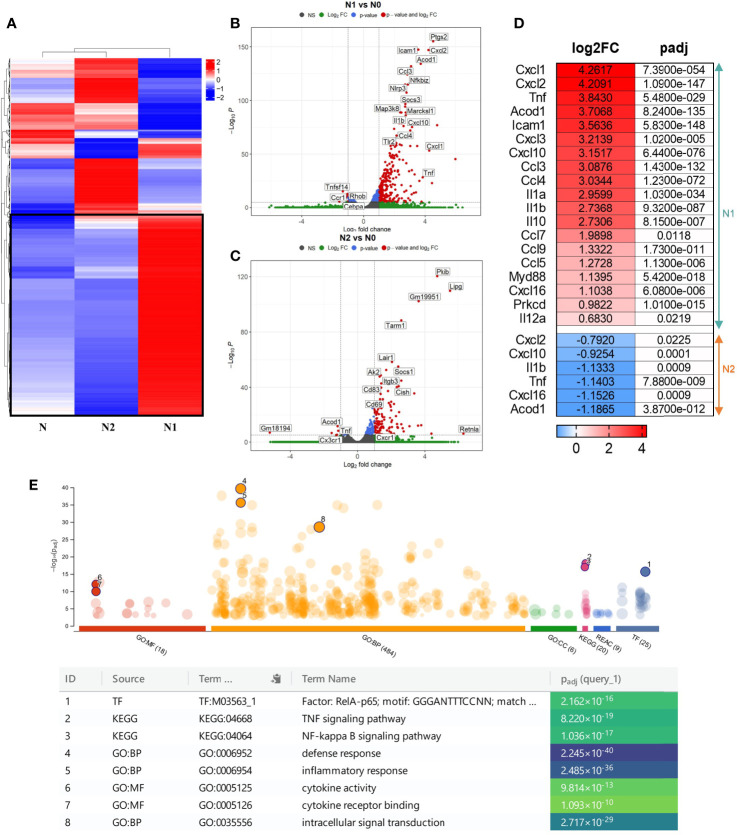
Gene expression profiling of the different neutrophil subsets. **(A)** Hierarchical Clustering Heatmap analysis of N, N1, and N2 neutrophils. Hierarchical clustering analysis was conducted of log2(FPKM+1) of differential expression genes union within all comparison groups. The color coding indicates different levels of expression: red indicates genes with high expression, and blue indicates genes with low expression levels. A major cluster of DEGs up-regulated in N1 is highlighted in a black square. **(B, C)** Volcano plot of differential gene expression between N1/N2 and N cells. The red dots represent significantly up-regulated and down-regulated genes with – adjP < 0.05 and Log2FC > 1: 771 genes were up-regulated and 195 genes were down-regulated in N1 *vs* N neutrophils; 532 genes were up-regulated and 124 genes were down-regulated in N2 *vs* N cells. **(D)** Heatmap showing log2 Fold change and adjusted p-value for selected inflammatory cytokines and chemokines differentially expressed either in N1 (upper panel) or N2 neutrophils (lower panel). Red color indicates the up-regulated, and blue the down-regulated genes. **(E)** Manhattan plot illustrating the results of the enrichment analysis of the gene cluster of highly up-regulated genes in N1 compared with N and N2 neutrophils. The functional terms are grouped and color-coded by data sources, i.e., molecular function (MF) in red, biological processes (BP) in orange, cellular components (CC) in green, KEGG in pink and TRANSFAC in dark blue. Numbered terms are detailed below the plot with their respective adjP values.

The hierarchical clustering analysis showed a distinct transcriptomic profile of the N1 and N2 neutrophil populations compared with control neutrophils (N) (**Figure 1A**). Additionally, we performed a differential expression analysis to generate modules of genes that are significantly modulated in each neutrophil population. With a cutoff criterion of absolute fold change ≥ 1.0 and adjP < 0.05, 966 genes were found to be differentially expressed in N1 neutrophils compared to control (771 genes were increased and 195 genes were decreased), and 532 genes were found to be differentially expressed in N2 neutrophils (408 genes were increased and 124 genes were decreased) (**Figures 1B, C**). In N1 neutrophils, a substantial number of up-regulated genes code for inflammatory cytokines and chemokines such as TNF-α, IL-10, IL-12, IL-1β, IL-1α, CCL3, CCL4, CCL5, CCL7, CCL9, CXCL1, CXCL2, CXCL3, CXCL10, CXCL16 (**Figure 1D**). These molecules were either unmodified in N2 neutrophils or were down-regulated compared to controls (TNF-α, IL-1β, CXCL16, CXCL2, CXCL10) (**Figure 1D**).

The gene ontology (GO) enrichment analysis highlighted biological processes and functions that are significantly associated with the modified genes in N1 and N2 neutrophils. The differentially modulated genes in N1 neutrophils are associated with cytokine production, cell response to LPS, cell chemotaxis and cytokine mediated-signaling pathways (**Figure S2A**). In contrast, genes found to be modified in N2 cells are related to T cell activation and differentiation, cell-cell adhesion and immune response (**Figure S2B**).

Interestingly, a central cluster of 391 highly up-regulated genes is well represented in N1 neutrophils compared with N2 and control cells (**Figures 1A** and **S3**). Functional enrichment analysis for the genes in this cluster revealed as significantly enriched the following terms: i) biological process - “defense response”; ii) molecular function - “cytokine activity” and “cytokine receptor binding”; iii) KEGG pathway analysis - “TNF signaling pathway”, “NF-kappa B signaling pathway”; iv) TRANSFAC - Factor: RelA-p65 as the most enriched transcription factor (**Figure 1E**). The results emphasize that the highly up-regulated genes in the N1 subset are associated with an inflammatory response. A list of the 10 most enriched terms for each database searched is presented in **Figure S4**.

**Figure 2:**
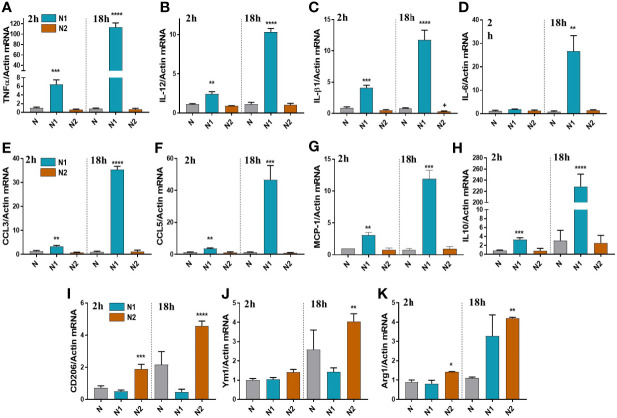
Gene expression of inflammatory and anti-inflammatory markers in neutrophils polarized for 2 or 18h with LPS+IFN*γ* (N1) or IL-4 (N2). **(A–G)** qPCR for inflammatory markers: TNFα, IL-12, IL-1β, IL-6, CCL3, CCL5, MCP-1, in N1 and N2 compared to control neutrophils (N). **(H–K)** qPCR for anti-inflammatory markers: IL-10, CD206, Ym1 and Arg1, in N1, N2 and N. n = 5, *p < 0.05, **p < 0.01, ***p < 0.001, ****p < 0.0001 (N1 or N2 *vs*. N).

Following the RNA-seq analysis, we validated a selection of differentially expressed inflammatory/anti-inflammatory genes by qPCR in neutrophils polarized for 2h or 18h. Compared to control neutrophils, the N1 neutrophils exhibited higher gene expression of the pro-inflammatory mediators TNF-α, IL-12, IL-1β, CCL2 (MCP-1), CCL3 (MIP-1α), and CCL5 (RANTES) both at 2h and 18h (**Figures 2A–G**). The gene expression of inflammatory cytokines was time-dependent, reaching higher levels at 18h compared with 2h of activation. IL-6 was highly induced in N1 neutrophils only after 18h of activation (**Figure 2D**). The anti-inflammatory markers CD206, Ym1 and Arg1 were increased in N2 neutrophils, but not in N1 cells (**Figures 2I–K**). However, the IL-10 gene expression was unchanged in N2 neutrophils and was overexpressed in N1 cells (**Figure 2H**). The data are in agreement with the results of RNA-seq expression where the gene encoding for IL-10 was found to be ~10-fold upregulated in N1 neutrophils.

Expression of inflammatory genes in N2 neutrophils was similar to unstimulated controls, except for the IL-1β that was decreased (**Figure 2C**). These results are in agreement with the results obtained by RNA-seq, where IL-1β and TNF-α were significantly decreased in N2 neutrophils compared with controls.

To validate these findings in an inflammatory state in humans, we analyzed the gene expression of CCL3, IL-6, IL-1β, and CD206 in human blood neutrophils isolated from MI patients during the first 24h after infarction. We found that the gene expression of CCL3 and IL-6 was significantly increased in neutrophils from these patients compared with healthy controls (**Figure S6**). These data demonstrate the involvement in human pathology of N1-like inflammatory neutrophils with a gene expression profile similar to the N1 neutrophils isolated from the infarcted myocardium (11) and to the N1 neutrophils derived *in vitro*. The gene expression of the N2 marker CD206 was not significantly affected in this early stage of the disease.

Additionally, we investigated the expression of N1/N2 surface markers by flow cytometry in a mouse model of endotoxemia (LPS-induced acute systemic inflammation). Circulating neutrophils were analyzed at 24h after the LPS treatment. Neutrophils from these mice were characterized by a significantly higher surface expression of CD11b (Mac-1) and ICAM-1 (**Figures S7D, E**), molecules involved in neutrophil adhesion, rolling and recruitment into the tissue. These results offer *in vivo* support for our *in vitro* data: RNA-seq data where ICAM-1 expression was 11 times increased in N1 compared with control neutrophils (log2FC:3,56; **Figure 1D**), cytokine array showing the increased shedding of sICAM-1 in N1 neutrophils (**Figures 4A, B**), and with the increased chemotactic activity of N1 neutrophils (**Figure 5G**). Similar to MI patients, LPS treatment did not modulate the surface CD206 expression of mice circulating neutrophils (**Figures S7D, E**), suggesting that N2-like neutrophils are not present in blood during the inflammatory stage.

### S100A9 Blockade Decreases Gene Expression of the Myeloid Chemotactic Chemokines CCL2, CCL3 and CCL5 in N1 Neutrophils

To assess the importance of S100A8/A9 for the immunostimulatory function of N1 neutrophils, we polarized bone marrow-derived neutrophils with LPS and IFN*γ* in the presence of the specific S100A9 blocker ABR-238901 (ABR). ABR-238901 inhibits the binding of S100A9 to its cognate receptors, as previously described (17).

**Figure 3:**
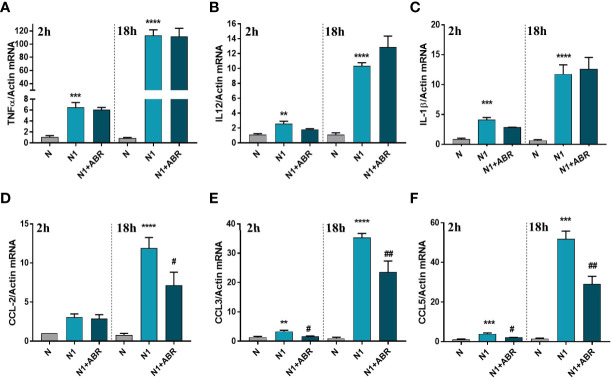
The effect of S100A9 blockade on gene expression of immune mediators **(A–F)** in neutrophils exposed to LPS+IFN*γ* (N1) or IL-4 (N2) in the presence of ABR-238901 (ABR). The treatment decreases significantly the gene expression of CCL2, CCL3 and CCL5 in N1 neutrophils at both 2h and 18h. n = 3; **p < 0.01, ***p < 0.001, ****p < 0.0001 (N1 *vs* N); ^#^p < 0.05, ^##^p < 0.01 (N1+ABR *vs* N1).

Gene expression of the inflammatory cytokines TNFα, IL-12, and IL-1β, and of the chemokines CCL2 (MCP-1), CCL3 (MIP-1α), and CCL5 (RANTES) was measured after 2h and 18h of stimulation. We found that S100A9 blockade significantly reduces the expression of CCL2, CCL3, and CCL5 (**Figures 3D–F**), but has no effect on the gene expression for inflammatory cytokines (**Figures 3A–C**).

### Soluble Immune Mediators Released by N1 and N2 Neutrophils

To confirm the mRNA results on protein expression level, we evaluated the levels of cytokines and chemokines present in the condition media of N1/N2 neutrophils after 18h polarization with LPS and IFN*γ*, with or without ABR-238901. The levels of inflammatory molecules were measured by a Proteome Profiler Mouse Cytokine array kit or by ELISA.

**Figure 4:**
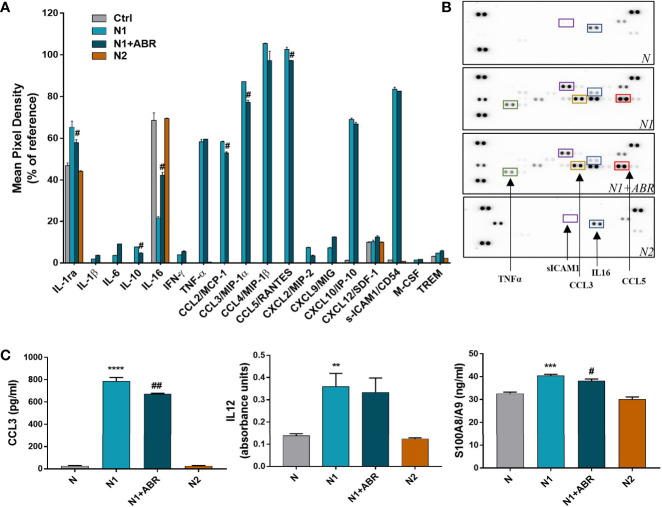
Effects of S100A9 blockade on neutrophil mediators released in the conditioned media after 18h of culture. **(A)** Quantification of mediators present in the culture medium from N1, N1 treated with ABR-23901, and N2 neutrophils compared to control neutrophils (N), as detected by the Proteome Profiler mouse cytokine array. The culture medium was pooled from three experiments. Protein levels were normalized to references on each membrane. ^#^p < 0.05 (N1+ABR versus N1 neutrophils). **(B)** Representative membranes incubated with the condition media from N, N1, N1+ABR and N2 neutrophils. **(C)** Measurement of CCL3, IL-12 and S100A8/A9 in the neutrophil s condition media by ELISA. The data represent mean ± SD from three experiments; **p < 0.01, ***p < 0.001, ****p < 0.0001 (N1 *vs* N); ^#^p < 0.05; ^##^p < 0.01 (N1+ABR *versus* N1).

The cytokine protein profiler was assessed in the neutrophil condition media pooled from 3 independent experiments. We found that control neutrophils secrete only 6 of the 40 cytokines determined by the kit, namely, IL-1ra, sICAM-1, IL-16, CXCL10, SDF1 and TREM. Compared with controls, N1 neutrophils secreted higher levels of 18 inflammatory cytokines/chemokines including CCL2, CCL3, CCL5, TNF-α (>30-fold change), as well as IL-6 and IL-1β (>2-fold change) (**Figures 4A, B**). S100A9 blockade significantly decreased the secretion of IL-1ra, IL-10, CCL2, CCL3, CCL5 and increased the production of IL-16. The inhibitory effects of the treatment on chemokine secretion agree well with the gene expression data (**Figure 3**).

To further verify the gene and protein array data, we have used ELISA to quantify the levels of a mediator that was modulated by ABR (CCL3) and one that was not (IL-12). The levels of both molecules were significantly higher in the N1 culture medium compared with control neutrophils, but S100A9 inhibition only reduced the secretion of CCL3 (**Figure 4C**). Additionally, we found a significantly higher S100A8/A9 secretion by N1 neutrophils compared to unstimulated controls and N2 neutrophils (**Figure 4C**), which was reduced by the ABR-238901 treatment. Taken together, the results suggest that S100A8/A9 promotes the secretion of chemotactic factors by N1 neutrophils, and that ABR-238901 has a dual inhibitory effect on S100A8/A9 secretion and function.

### Functional Assessment of the N1 and N2 Neutrophil Subtypes

#### Production of ROS and NO

**Figure 5:**
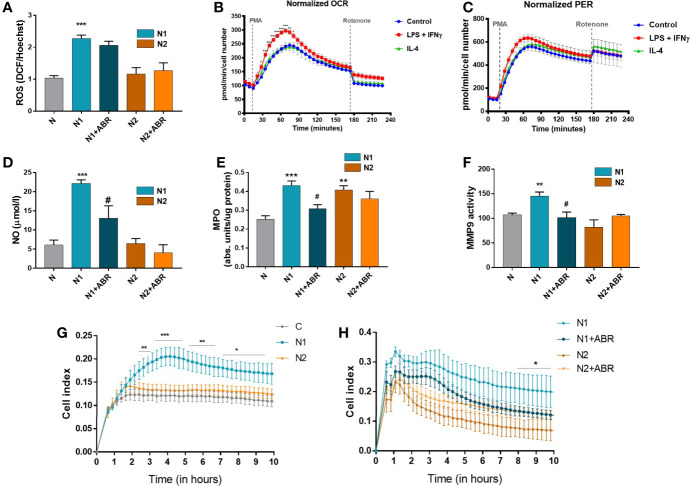
Functional analysis of neutrophil populations. The cells were treated for 18h with LPS + IFN*γ* (N1) or IL-4 (N2) in the presence or absence of ABR-238901 (ABR). **(A)** Quantification of intracellular ROS using the DCFDA assay. **(B, C)** Measurement of the neutrophil oxidative burst evaluated in response to phorbol 12-myristate 13-acetate (PMA): **(B)**the oxygen consumption rate (OCR) and **(C)** the glycolytic proton efflux rate (PER), calculated using extracellular acidification rate (ECAR) measurements. **(D)** Quantification of NO released in the conditioned medium of neutrophil subtypes. **(E)** evaluation of MPO enzymatic activity in the cell lysate of neutrophil populations. **(F)** Quantification of MMP-9 gelatinase activity assessed by SDS‐PAGE zymography in the condition media of neutrophils. **(G, H)** The chemotactic activity of neutrophil populations toward IL-8 evaluated using a CIM-plate 16 and the xCELLigence RTCA DP system. The cell index (CI), proportional to the number of transmigrated neutrophils, was measured as the cell electrical impedance every 15 min over 10h. Data are from four independent experiments; every experiment used pooled neutrophils from at least 5 mice. Data are shown as mean fold change to control ± SE (n = 4). Statistical significance is shown as *p < 0.05, **p < 0.01, ***p < 0.001, ^#^p ≤ 0.05.

It has previously been shown that stimulated neutrophils activate NADPH oxidase (NOX2) to generate large amounts of superoxide, which acts as a precursor of hydrogen peroxide and other ROS (30). Intriguingly, factors that stimulate the oxidative burst might also simultaneously trigger iNOS activation in neutrophils or vice versa (31). We set up experiments to compare the capacity of N1 and N2 neutrophils to produce ROS and NO, and to determine whether S100A9 blockade influences these processes. We found that, in contrast with the N2 subtype, N1 neutrophils have significantly higher levels of ROS and NO compared to controls (**Figures 5A, D**). The S100A9 inhibition significantly reduced NO production in N1 neutrophils but did not affect ROS levels in either population.

#### The Energetic Profile of N1 and N2 Neutrophils

Cellular metabolism plays a decisive role in the function and plasticity of immune cells (1). Since the effector functions of neutrophils during inflammation are tightly linked to their metabolic state, we have investigated the energetic changes occurring in the N1 and N2 populations. The oxidative burst of neutrophils in basal conditions (control) or the presence of LPS + IFN*γ* (N1) or IL-4 (N2) was quantified by measuring the oxygen consumption rate (OCR) in response to phorbol 12-myristate 13-acetate (PMA). We found an increased OCR by N1 neutrophils compared to controls or N2. The increase in OCR or oxidative burst after activation with PMA was associated with a simultaneous increase in the Proton Efflux Rate (PER), indicating that neutrophils depend on glycolysis for activation (**Figures 5B, C**). Exposure to LPS+IFN*γ* increased the glycolytic function of neutrophils, which were transformed into metabolically less efficient cells. Inhibition of mitochondrial respiration with rotenone revealed that mitochondria only have a modest contribution to neutrophil oxidative burst after PMA activation (**Figures 5B, C**).

#### MPO and MMP-9 Activity in Polarized Neutrophils

MPO and MMP-9, along with elastase, are the main tissue destructive enzymes produced by neutrophils and are involved in matrix and protein degradation. We measured the activity of MPO in the cell lysate and of MMP-9 released in the condition media collected from N1 and N2 neutrophils. As shown in **Figure 5E**, MPO activity was significantly increased in both neutrophil phenotypes compared to control. In contrast, MMP-9 activity was only augmented in N1 neutrophils (**Figure 5F**). S100A9 inhibition with ABR-238901 restored both MPO and MMP-9 activity in N1 neutrophils to levels similar to the unstimulated control (**Figures 5E, F**).

#### Transmigration of Polarized N1 and N2 Neutrophils

The transmigration capacity of polarized neutrophils was monitored using the chemokine IL-8 as a chemoattractant. Despite the lack of a gene coding for IL-8, mice express a receptor homologous to human CXCR2 that mediates neutrophil chemotaxis in response to human IL-8 (32). Neutrophils were placed in the upper chamber of a CIM-plate, and RPMI culture medium containing IL-8 was added to the lower chamber. The chemotactic activity was monitored over 20h using the xCELLigence software. As shown in **Figure 5G**, N1 neutrophils exhibited significantly increased migration capacity compared with N2 or control neutrophils. Only a few neutrophils transmigrated in the lower chamber when RPMI without IL-8 was used as a negative control (not shown). The addition of ABR-238901 in the upper chamber inhibited the migratory capacity of N1 neutrophils (**Figure 5H**).

### Molecular Players Involved in the Function of Polarized Neutrophils

#### NADPH Oxidase Subunits Regulate ROS Production in Neutrophils

**Figure 6:**
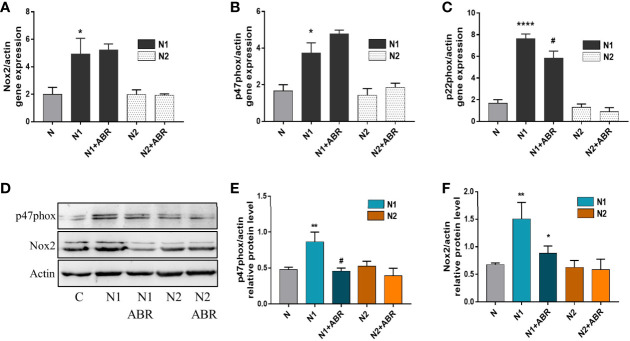
Gene and protein expression of NADPH oxidase subunits in the different neutrophil populations in the presence or absence of ABR-238901 (ABR). **(A-C)** Quantification of gene expression for NADPH oxidase subunits Nox2, p47phox and p22phox by qPCR. **(D-F)** Protein expression of NADPH oxidase subunits p47phox and Nox2 determined by Western blot. n = 3, *p < 0.05, **p < 0.01 ****p < 0.0001 (N1 *versus* control); ^#^p<0.05 (N1 *versus* N1 + ABR).

Upon activation, neutrophils produce large amounts of ROS *via* the NADPH oxidase complex (33). To investigate the mechanisms responsible for the differences in ROS production between the N1 and N2 neutrophils, we assessed the expression of the main subunits of NADPH oxidase complex in both neutrophil subtypes by real-time PCR and Western blot. Gene expression of Nox2, p47phox and p22phox was significantly increased in N1 neutrophils compared to control (**Figure 6A–C**). Inhibition of S100A9 significantly reduced gene expression of the p22phox subunit. Protein expression of Nox2 and p47phox was also increased in N1 cells. Interestingly, the treatment of polarized neutrophils with ABR-238901 significantly reduced p47phox and Nox2 protein level in N1 neutrophils (**Figure 6D–F**), although no effect was observed at gene expression level (**Figure 6A, B**). Expression of the NADPH subunits in N2 neutrophils was similar to controls on both gene and protein levels and was not influenced by the S100A9 blockade.

#### Signaling Pathways Activated in N1 and N2 Polarized Neutrophils

**Figure 7:**
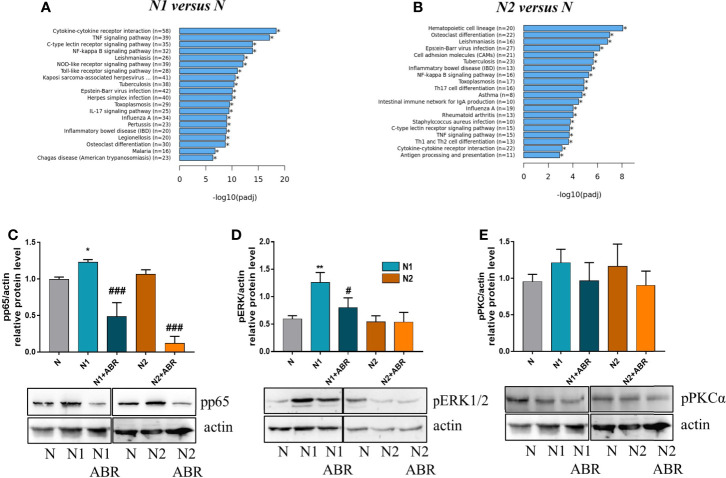
Signaling pathways activated in neutrophils subtypes. **(A, B)** The top 20 signaling pathways revealed by the KEGG signaling pathway enrichment analysis in N1 and N2 compared with control neutrophils (N). **(C-E)** Analysis of the phosphorylation form of p65, ERK and PKC in neutrophil subsets. Cell lysate from control (N), or polarized neutrophils (N1 and N2) cultured for 18h in the presence or absence of ABR-238901 (ABR) was analyzed by Western blot; n = 3, *p < 0.05, **p < 0.01 (N1 *vs* N), ^#^p < 0.05, ^###^p < 0.001 (N1 *vs* N1 + ABR).

RNA-seq analysis identified over 20 signaling pathways that were significantly enriched in N1 and N2 neutrophils [-log10(p adj)>5] (**Figures 7A, B**), with NF-kB signaling pathway comprising over 30 DEGs (**Figure 7A**). Among the transcription factors, RelA-p65 was the most up-regulated in N1 neutrophils compared to control (adjP: 2.162 x 10^-16^) (**Figure 1E**). To validate these results, we compared the activation status of the NF-kB signaling pathway in N1 *versus* N2 neutrophils. Phosphorylated p65 was significantly increased in N1 neutrophils and unchanged in N2 cells compared with unstimulated control, as assessed by Western Blot (**Figure 7C**). We also evaluated the activation of two other signaling molecules associated with inflammation and oxidative burst, ERK1/2 and PKC. We found a significant increase in pERK1/2 protein expression, but no differences in pPKC levels (**Figures 7D, E**). Blockade of S100A9 with ABR-238901 significantly inhibited both pERK and pp65, supporting its anti-inflammatory properties. pPKC was not modified by ABR-238901 in any of the neutrophil subtypes (**Figure 7E**).

## Discussion

Neutrophils, the most abundant leukocytes in human blood, are the first innate immune effectors in infections and sterile inflammation. Aberrant neutrophil responses are associated with various diseases such as sepsis, asthma, MI, and rheumatoid arthritis (34, 35). Recent data have shed new light on the neutrophil responses in various pathologies and the classical view that neutrophils are a homogeneous population has been revised following the identification of novel functions and phenotypic diversity (33, 34). Pro-inflammatory (N1) and anti-inflammatory (N2) neutrophils have been identified under various pathological conditions *in vivo* (2, 11, 36). Recently, Ma et al. have described the sequential infiltration of N1 and N2 neutrophils in infarcted mouse hearts and demonstrated that these cells share phenotypic features with N1 neutrophils derived *in vitro* from naïve neutrophils in the presence of LPS/IFN*γ* and with IL-4 derived N2 neutrophils (11).

The purpose of our study was to examine the functional differences between the N1 and N2 subpopulations, and to assess the importance of the abundant neutrophil mediator S100A8/A9 in promoting these phenotypes. We have therefore used the same *in vitro* protocol employed by Ma et al. (11). We found that N1 and N2 neutrophil populations have distinct transcriptomic profiles and functions. N1 neutrophils exhibit increased production of inflammatory cytokines/chemokines, elevated levels of ROS and NO, augmented oxidative burst, increased activity of protein and matrix-degrading enzymes, as well as enhanced chemotactic response. Conversely, N2 neutrophils display increased expression of CD206, Ym1 and Arg1, and have similar ROS and NO levels, oxidative burst and chemotactic response as the unstimulated controls. Further, we found that the phosphorylated forms of ERK1/2 and p65, signaling molecules associated with an inflammatory phenotype, are increased in N1 but not in N2 neutrophils. Finally, S100A8/A9 blockade lowered the phosphorylation of ERK1/2 and p65 in N1 neutrophils, leading to reduced production of the chemokines CCL2, CCL3, CCL5, reduced NO, MPO and MMP-9 activity, and slower N1 migration. These data are all the more important as *in vivo* we found that similar N1 inflammatory neutrophils are present in a human inflammation state (post-MI patients), as well as in a mouse model of endotoxemia.

Our RNA-seq analysis of the transcriptomic profile of the two neutrophil populations polarized *in vitro* revealed that N1 neutrophils overexpress genes associated with cytokine production, chemotaxis and cytokine mediated-signaling pathways involved in pro-inflammatory responses. In contrast, the N2 gene profile includes genes involved in T cell activation and differentiation, cell-cell adhesion and other immune responses. Our data confirm the previously described expression of CCL3, CCL5, IL-12a, and TNF-α in N1 neutrophils (11), and identify additional inflammatory chemokines and cytokines up-regulated in this population, such as IL-1α, CCL4, CCL7, CCL9, CXCL1, CXCL2, CXCL3, CXCL10, and CXCL16. Importantly, we found that IL-16 was down-regulated in N1 neutrophils, and TNF-α and IL-1β were down-regulated in N2 neutrophils compared with controls. IL-16 has previously been found to be stored in the neutrophil cytosol and released under conditions of insufficient clearance of apoptotic neutrophils that typically occur at sites of infection and inflammation (37). However, the biological significance of the lower IL-16 release from N1 neutrophils found in our study remains unclear.

Our study is the first to identify that the alarmin S100A8/9, abundantly secreted by activated neutrophils, is an important promoter of the aggressive pro-inflammatory N1 phenotype through an autocrine mechanism. We show that N1 neutrophils secrete higher amounts of S100A8/A9 compared to N2 cells, and that inhibition of S100A9 with the specific blocker ABR-238901 reduces the secretion of S100A8/A9 and of the myeloid chemoattractants CCL2, CCL3 and CCL5. These results extend previous data showing that S100A8/A9 modulates the production of pro-inflammatory mediators including cytokines, chemokines, ROS, and NO in various cell types (38). The reduced production of myeloid chemoattractants induced by S100A9 inhibition provide important mechanistic support to our previous findings in a mouse model of MI *in vivo*, showing that treatment with ABR-238901 prevents neutrophil and monocyte migration from the bone marrow and spleen into the circulation, and recruitment into the heart (17, 39). Consequently, the treatment reduced myocardial inflammation and significantly improved cardiac function compared to controls (17). A close examination of the myocardial environment showed reduced S100A8/A9 staining and CCL5 gene expression at the end of the 3-day treatment (17), which is in agreement with the *in vitro* data presented here. Our findings support the important role of S100A8/A9 as a pro-inflammatory neutrophil mediator, adding to previous data showing that S100A8/A9 induces neutrophil release from the bone marrow and directs their migration in response to LPS (40), primes the NLRP3 inflammasome in neutrophils and stimulates IL-1β production post-MI (41), and is indispensable for MI-induced granulopoiesis (18). Interestingly, in the presence of extracellular calcium the S100A8/A9 heterodimers form inactive (S100A8/A9)_2_ tetramers that prevent excessive systemic pro-inflammatory effects (42). S100A9 blockade did not have any consequences on N2 neutrophil phenotype and function evaluated in this study. However, we have previously shown that S100A8/A9 activates the transcription factor Nur77 in monocytes and promotes the generation of MerTK^hi^ reparatory macrophages (39). These data add further evidence for the complex activity of these proteins, depending on their biological form, cell type and disease stage.

At the site of infections, activation of NOX2 in neutrophils generates large amounts of ROS, which are essential for antimicrobial host defense (30). However, excessive ROS production also induces tissue injury and exacerbation of the inflammatory reaction in different pathologies. In MI, excessive ROS production by neutrophils may damage the healthy myocardium and promote ventricular remodeling (43). We found that the level of ROS was significantly higher in N1 neutrophils, which have also been shown to dominate the pro-inflammatory phase of the immune response in MI (11). Inhibition of S100A9 significantly decreased the protein levels of the NADPH oxidase subunits Nox2 and p47 in N1 neutrophils, but the gene expression was unaffected. Previous studies have found that S100A8/A9 heterodimer interacts directly with the cytosolic phox proteins p67 and p47phox (44) and with Nox2 (45) and induces ROS production by activating NADPH oxidases (45, 46). This interaction might explain the observed effects of S100A9 inhibition in our study, but the exact mechanisms remain to be elucidated.

We also found that N1 neutrophils migrate in higher numbers toward IL-8 compared with N2 or control neutrophils, and S100A9 inhibition with ABR-238901 significantly reduced N1 neutrophil transmigration. These data are in good agreement and extend previous results reporting that S100A8, S100A9, and S100A8/A9 are involved in neutrophil migration to inflammatory sites (47), and that leukocyte migration is deficient in S100A9-knockout mice (48). Limiting neutrophil chemotaxis by S100A9 blockade could be a therapeutic strategy for pathologies where excess neutrophil infiltration and activation cause inflammation, impair tissue repair and lead to loss of organ function. Neutrophils mediate tissue damage through the release of proteases from their cytoplasmic granules. As expected, we found that inflammatory N1 neutrophils release an increased level of active MMP-9 and MPO. Exposure of N1 neutrophils to ABR-238901 reduces the activity of both enzymes to levels similar to control cells, adding support to the possible importance of S100A9 blockade in limiting tissue damage.

Understanding the signaling mechanisms involved in the production and release of cytokines/chemokines, proteases, and ROS from neutrophils could provide novel targets for anti-inflammatory therapies. Both LPS and S100A8/A9 activate the TLR4/MD2 receptor complex, leading to recruitment of the adaptor protein MyD88 and sequential activation of IRAK1, ERK, p38 MAPK, and NF-κB (38). S100A8/A9 binding to RAGE also leads to NF-κB activation (14, 38). Here, we show that the MyD88 gene is significantly upregulated and the phosphorylated forms of ERK1/2 and the p65 subunit of NF-kB are more abundant in N1 neutrophils compared to controls. These pathways mediate the neutrophil response to aggressors, including ROS production, cytokine and chemokine synthesis, and induction of anti-apoptotic signals (49). S100A9 blockade reduced the phosphorylation of both p65 and ERK1/2, demonstrating an important contribution of the protein in triggering these pathways. Since both externally-supplied LPS and neutrophil-secreted S100A8/A9 are present in our *in vitro* system, it is difficult to distinguish the relative contribution of the two mediators in triggering TLR4 activation, as they compete for the receptor. The magnitude of the observed effects of S100A9-blockade on N1 neutrophils varied depending on the outcome. The treatment led to important inhibition of N1 migratory ability, NO, MPO and MMP-9 secretion, but only modest reduction in cytokine and chemokine secretion. These results suggest that only certain pathways are affected by the S100A9 inhibition in our system. One possibility could be that ABR-238901 only interferes with the activation of RAGE, while S100A8/A9 binding to TLR4/MD2 is prevented by the presence of LPS. However, this hypothesis is highly speculative and requires confirmation in future experiments employing different combinations of TLR4 and RAGE blockers or LPS-free experimental systems.

Recently, it has been demonstrated that the absence of TLR4 in a mouse model of stroke polarizes the cells toward an N2 phenotype associated with neuroprotection (50). The data suggest a different modulation of neutrophils in absence of TLR4. In N2 neutrophils we found an increased expression of anti-inflammatory markers and reduced production of the inflammatory cytokines TNF-α and IL-1β. Functionally, these cells were similar to control neutrophils in our experimental setting. However, the N2-like phenotype obtained by IL-4 stimulation *in vitro* may differ from the anti-inflammatory neutrophils found *in vivo*. While the N1 inflammatory phenotype appears to dominate the acute response to infection or inflammation *in vivo*, a complex microenvironment is likely to lead to higher neutrophil diversity after the acute phase has ended. This assumption is supported by a recent report investigating cardiac neutrophil diversity in murine MI. The study demonstrated the existence of temporal diversity of neutrophil states in the infarcted heart, identifying 6 transcriptionally distinct cell clusters with a time-dependent appearance (51). In a study focused on tumor-associated neutrophils, the authors also used LPS and IFN*γ*/IFNβ to derive N1 neutrophils and a much more complex mediator cocktail to derive N2 cells. The cocktail included L-lactate, adenosine, TGF-β, IL-10, prostaglandin E2, and G-CSF, in an attempt to mimic the tumor environment (52). The resulting N2 neutrophils are likely to differ from the IL-4-derived neutrophils used in the present study.

### Study Limitations

Our study has several important limitations that have to be considered when interpreting the findings. Firstly, it is important to acknowledge that our simplified experimental system is unlikely to fully reproduce the complex environment present *in vivo*. We chose to employ the same *in vitro* conditions proposed by Ma et al., as these have been shown to generate N1/N2 neutrophils similar to the cells found by these authors in the infarcted mouse hearts *in vivo*. However, as mentioned above, a more detailed study of neutrophil genetic profile post-MI has identified 6 distinct cell clusters that sequentially infiltrated the post-ischemic myocardium (51). It remains unclear whether and how these cells will be able to be generated *in vitro* with enough fidelity in future studies. Secondly, as discussed above, LPS and S100A8/A9 are competing for the TLR4 receptor, which makes it difficult to discern to what extent the two mediators contribute to the observed effects. Lastly, ABR-238901 has specifically been developed to bind to S100A9 and inhibit activation of TLR4 and RAGE. Quinoline-3-carboxamides, first-generation S100A9 blockers, have been shown to block the binding of both the S100A9 homodimer and of the S100A8/A9 heterodimer to mouse and human TLR4 and RAGE (53). However, it has not been tested whether the next-generation blocker ABR-238901 is also able to inhibit the binding of both forms of the protein to the receptors. Therefore, we cannot determine with certainty whether the observed effects are solely due to S100A9 blockade or to the blockade of both forms of the protein.

In conclusion, our study contributes to the understanding of the transcriptomic, phenotypic and functional characteristics N1 and N2 neutrophils and is the first to identify an important autocrine role of the neutrophil mediator S100A8/A9 in promoting the pro-inflammatory N1 phenotype. These data support previous results suggesting a pathogenic role of S100A8/A9 in clinical trials and *in vivo* models, and promote pharmacological blockade of S100A9 as a potentially important therapeutic strategy in inflammatory disorders with a neutrophil component.

## Data Availability Statement

The datasets presented in this study can be found in online repositories. The names of the repository/repositories and accession number(s) can be found below: https://www.ebi.ac.uk/arrayexpress/E-MTAB-10508.

## Ethics Statement

The studies involving human participants were reviewed and approved by Ethics Committee of Elias University Emergency Hospital Bucharest. The patients/participants provided their written informed consent to participate in this study. The animal study was reviewed and approved by The National Sanitary Veterinary and Food Safety Authority (nr. 425/22.10.2018), the Institutional Ethics Committee of ICBP “N. Simionescu”, and by the Regional Ethics Committee for Animal Research in Lund, Sweden.

## Author Contributions

EBu contributed to conception and design of the study, wrote the first draft of the manuscript and performed revision. AS and MS contributed to conception of the study and to manuscript revision. RDM performed RNA-seq analysis and organized the database. ACM, LC, SC, MV, AMG, AH, MMT isolated neutrophils from bone marrow and performed all the experiments. BP designed the RNA-seq experiment and Seahorse assay. ACM and EBu performed the statistical analysis. LC wrote sections of the manuscript. GJ performed experiments with in vivo mouse model of inflammation. EBa and SB recruted the MI patients, isolated blood and managed the ethical approval for research involving human participants. All authors contributed to the article, read and approved the submitted version.

## Funding

This work was supported by a grant of Ministry of Research and Innovation, CNCS - UEFISCDI, project number PN-III-P4-ID-PCCF-2016-0172, within PNCDI III” and by the Romanian Academy. AS and GJ are also supported by grants from the Swedish Heart and Lung Foundation and from the Swedish Research Council (Vetenskapsrådet).

## Conflict of Interest

The authors declare that the research was conducted in the absence of any commercial or financial relationships that could be construed as a potential conflict of interest.

## Publisher’s Note

All claims expressed in this article are solely those of the authors and do not necessarily represent those of their affiliated organizations, or those of the publisher, the editors and the reviewers. Any product that may be evaluated in this article, or claim that may be made by its manufacturer, is not guaranteed or endorsed by the publisher.
