# Supplementary material for: Transcriptional Profiling and Functional Analysis of N1/N2 Neutrophils Reveal an Immunomodulatory Effect of S100A9-Blockade on the Pro-Inflammatory N1 Subpopulation
Source: Front Immunol. 2021 Aug 10;12:708770. doi: 10.3389/fimmu.2021.708770 (PMC8384118; doi:10.3389/fimmu.2021.708770)
Supplement: Supplementary file 1 [file DataSheet_1.docx]

**Supplementary materials**

**Supplementary Methods**

**Isolation of human neutrophils**

Blood (5 ml) was collected from four MI patients within 24h after the acute ischemic event. Four healthy subjects were used as the control group. Neutrophils were isolated within the first 60 minutes after blood collection using the MACSxpress® Whole Blood Neutrophil Isolation Kit (Miltenyi). Purity was confirmed by flow cytometry using the neutrophil marker CD66. The study was approved by the Ethics Committee of the Elias University Emergency Hospital Bucharest, and the investigation was carried out according to the principles outlined in the Declaration of Helsinki (1). All participants gave written informed consent. The samples have been anonymized before analysis.

**Quantitative RT-PCR**

Total RNA was isolated from 10x10^6^ neutrophils from each patient or control, using the TRIzol reagent. First-strand cDNA synthesis was performed employing 1 μg of total RNA and MMLV reverse transcriptase, according to the manufacturer's protocol (Invitrogen). Amplification of cDNA was performed using a LightCycler 480 Real-Time PCR System (Roche) and SYBR Green I. The human primer sequences for the mRNAs of interest are shown in Supplementary Table 4. The relative quantification was done by the comparative CT method and expressed as arbitrary units.

***In-vivo* mouse model of endotoxin-induced inflammation**

C57Bl/6 female mice, 8 weeks of age, were administered 5mg/kg LPS or PBS through intraperitoneal injection. Blood was harvested 24 hours later by cardiac puncture and 400-500 µL of blood was diluted with 3 mL HBSS containing 15mM EDTA on ice. The blood was centrifuged for 5 min at 400g and red blood cells were lysed with 5 mL of Ammonium Chloride lysis buffer for 5 minutes at room temperature. Lysis was halted with 10 mL HBSS containing 1% BSA and 2mM EDTA, followed by centrifugation for 5 min at 400g. Lysis was repeated until the cell pellets were free of red blood cells. The remaining cell suspension was washed twice with PBS before cell staining for flow cytometry.

**Flow Cytometry.**

Single-cell suspensions were stained for viability using Zombie/Aqua (BioLegend) for 20 minutes at 4°C, followed by extracellular staining in PBS supplemented with 1% BSA and 2mM EDTA for 30 minutes at 4°C, washing and analysis. The following monoclonal antibodies were purchased from BioLegend and used for flow cytometric analysis: anti-Ly-6G (1A8), anti ICAM-1 (YN1/1.7.4), anti-CD45.2 (104), anti CD11b (M1/70) and anti-CD206(C068C2). The samples were acquired on a Gallios (Beckman Coulter) using the Kaluza software (Beckman Coulter) and further analyzed using FlowJo (TreeStar).

**Supplementary Tables**

**Supplementary table 1**: Data quality summary

| QC_summary | |  |  |  |  |  |  |  |
| --- | --- | --- | --- | --- | --- | --- | --- | --- |
| Sample name | Raw reads | Clean reads | Raw bases | Clean bases | Error rate(%) | Q20(%) | Q30(%) | GC content(%) |
| N_1 | 36996096 | 36297606 | 11.1G | 10.9G | 0.02 | 98.01 | 94.42 | 50.23 |
| N_2 | 35761965 | 35108978 | 10.7G | 10.5G | 0.02 | 97.99 | 94.4 | 50.62 |
| N_3 | 39214939 | 38556498 | 11.8G | 11.6G | 0.02 | 98.06 | 94.52 | 50.32 |
| N1_1 | 35605508 | 34896927 | 10.7G | 10.5G | 0.02 | 98.1 | 94.64 | 50.94 |
| N1_2 | 34535577 | 33939546 | 10.4G | 10.2G | 0.02 | 97.93 | 94.27 | 50.77 |
| N2_1 | 35076480 | 34293200 | 10.5G | 10.3G | 0.03 | 97.83 | 94.08 | 50.55 |
| N2_2 | 37846131 | 37194740 | 11.4G | 11.2G | 0.02 | 98.31 | 95.08 | 51.03 |
| N2_3 | 34826931 | 34143759 | 10.4G | 10.2G | 0.02 | 98 | 94.41 | 50.4 |

**Supplementary table 2**: Mapping result summary

| MapStat_summary | |  |  |  |  |  |  |  |
| --- | --- | --- | --- | --- | --- | --- | --- | --- |
| Sample name | N_1 | N_2 | N_3 | N1_1 | N1_2 | N2_1 | N2_2 | N2_3 |
| Total reads | 72595212 | 70217956 | 77112996 | 69793854 | 67879092 | 68586400 | 74389480 | 68287518 |
| Total mapped reads | 69295696 | 66909466 | 73632410 | 65888100 | 63509968 | 62431316 | 71207242 | 65144904 |
| Uniquely mapped reads | 61742704 | 58177144 | 66407008 | 58762734 | 57433644 | 55661510 | 60757644 | 58630902 |
| Multiple mapped reads | 7552992 | 8732322 | 7225402 | 7125366 | 6076324 | 6769806 | 10449598 | 6514002 |
| Total mapping rate | 95.45% | 95.29% | 95.49% | 94.40% | 93.56% | 91.03% | 95.72% | 95.40% |
| Uniquely mapping rate | 85.05% | 82.85% | 86.12% | 84.19% | 84.61% | 81.16% | 81.68% | 85.86% |
| Multiple mapping rate | 10.40% | 12.44% | 9.37% | 10.21% | 8.95% | 9.87% | 14.05% | 9.54% |

**Supplementary table 3**: The mouse sequences of oligonucleotide primers used for evaluation of gene expression.

| **Gene  (Mus musculus)** | **Sequences of Oligonucleotide Primers** | **GenBank®  Accession Number** | **Predicted Size (bp)** |
| --- | --- | --- | --- |
| TNFα | GGTGCCTATGTCTCAGCCTCT | NM_013693.3 | 312 |
|  | CATCGGCTGGCACCACTAGTT |  |  |
| IL12a | CAATCACGCTACCTCCTCTTTT | NM_008351.3 | 181 |
|  | CAGCAGTGCAGGAATAATGTTTC |  |  |
| CCL5 | ACCACTGCCCTTGCTGTTCTTC | NM_011337.2 | 117 |
|  | TGGAATCTTCCGGCTGTAGGAG |  |  |
| CCL3 | TTCTCTGTACCATGACACTCTGC | NM_011337.2 | 100 |
|  | CGTGGAATCTTCCGGCTGTAG |  |  |
| Arg1 | AACAGGCAGTCGCTTTAACC | NM_007482.3 | 116 |
|  | GGTTTTCATGTGGCGCATTC |  |  |
| CD206(MRC1) | CAGGTGTGGGCTCAGGTAGT | NM_008625.2 | 118 |
|  | TGTGGTGAGCTGAAAGGTGA |  |  |
| IL10 | GCTCTTACTGACTGGCATGAG | NM_008625.2 | 105 |
|  | CGCAGCTCTAGGAGCATGTG |  |  |
| Ym1 | TGGAATTGGTGCCCCTACAA | NM_009892.3 | 144 |
|  | GCATAGGGTACTTCCTGGGG |  |  |
| IL 6 | CTGCAAGAGACTTCCATCCAG | NM_031168.2 | 131 |
|  | AGTGGTATAGACAGGTCTGTTGG |  |  |
| IL1β | GAAATGCCACCTTTTGACAGTG | NM_008361.4 | 116 |
|  | TGGATGCTCTCATCAGGACAG |  |  |
| CCL2 | TAAAAACCTGGATCGGAACCAAA | NM_011333.3 | 120 |
|  | GCATTAGCTTCAGATTTACGGGT |  |  |
| Nox2 | ACTCCTTGGGTCAGCACTGG | NM_007807.5 | 160 |
|  | GTTCCTGTCCAGTTGTCTTCG |  |  |
| p22 phox | TGGCCTGATTCTCATCACTGG  GGGACAACTCCACAGAAACTC | NM_007806.3 | 579 |
| p47 phox | GGGGCGATCAATCCAGAGAAC | NM_001286037.1 | 105 |
|  | GTACTCGGTAAGTGTGCCCTG |  |  |

**Supplementary table 4**: The human sequences of oligonucleotide primers used for evaluation of gene expression.

| **Gene  (homo sapien)** | **Sequences of Oligonucleotide Primers** | **GenBank®  Accession Number** | **Predicted Size (bp)** |
| --- | --- | --- | --- |
| CD206 | GGGTTGCTATCACTCTCTATGC |  |  |
|  | TTTCTTGTCTGTTGCCGTAGTT | NM_002438.4 | 126 |
| IL-1β | AGCTACGAATCTGCGACCAC |  |  |
|  | CGCTATCCCATGTGTTGAAGAA | NM_000576.3 | 186 |
| Il-6 | CTGCAGAATTCCAGGACCACA |  |  |
|  | TCCGGTGGTGTAAAGAGGAC | NM_000600.5 | 106 |
| CCL3 | AGTTCTCTGCATCACTTGCTG |  |  |
|  | CCGCTTCGCTTGGTTAGGAA | NM_002983.3 | 151 |

**Supplementary Figures**


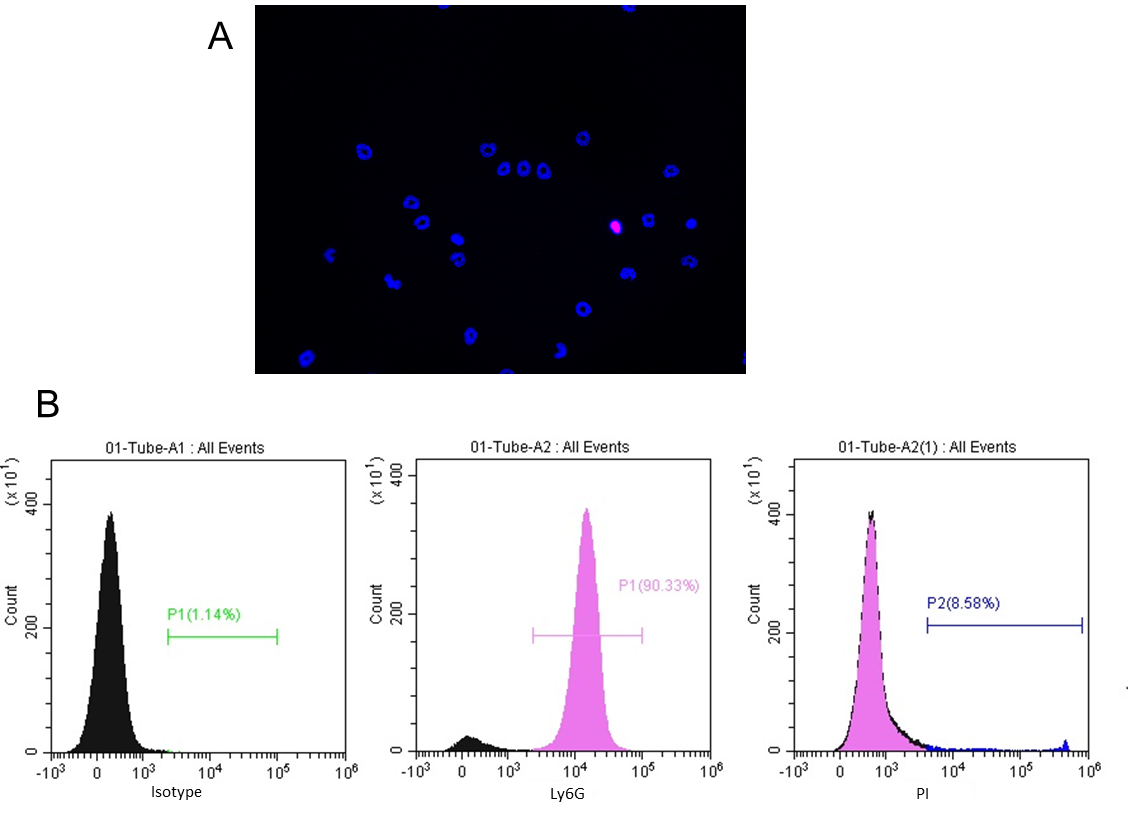


**Figure S1. Related to Methods 1**. Purity of isolated neutrophils. **A**. Hoechst 33342 and propidium iodide (PI) double staining of primary neutrophils isolated from bone marrow using percoll. Pink indicates the dead cells. Images were taken with 60× objective. B. Flow cytometry

analyses showing the number of neutrophils-labeled Ly-6G (90,33%), or dead cells, PI-positive (8.58%).


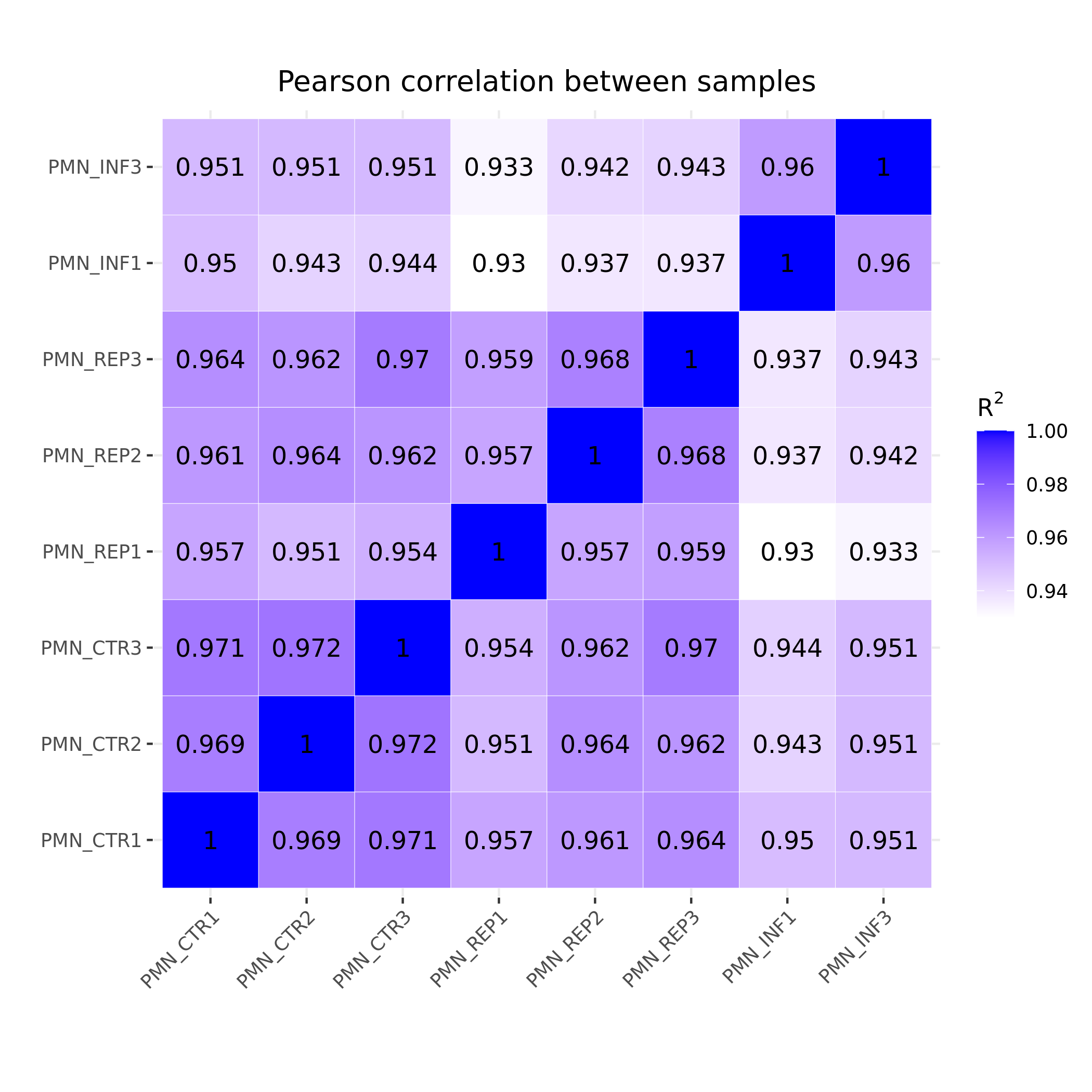


N_1

N_2

N_3

N2_1

N2_2

N2_3

N1_1

N1_2

N_1

N_2

N_3

N2_1

N2_2

N2_3

N1_1

N1_2

**Figure S2**. The Pearson correlation matrix show a high correlation between replicates (N samples minimum R² of 0.969; N1 samples with an R² of 0.96 and N2 replicates with a minimum R² 0.957).


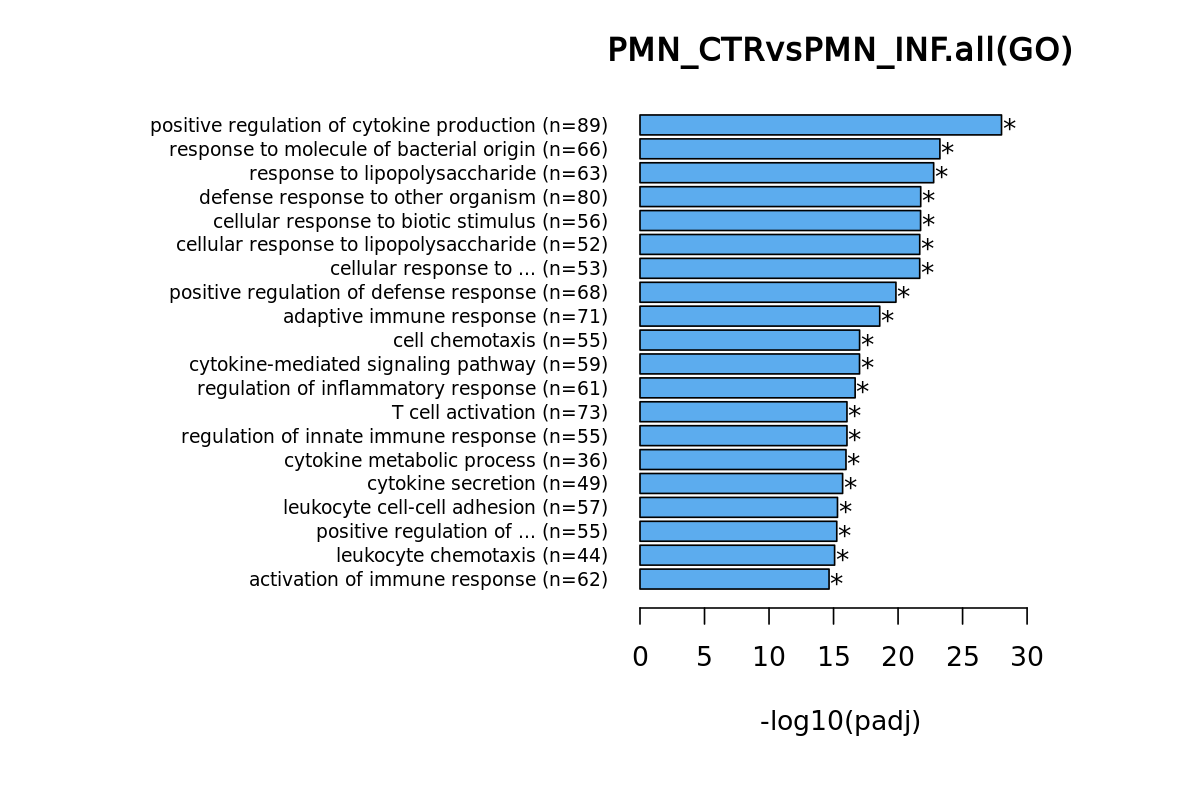

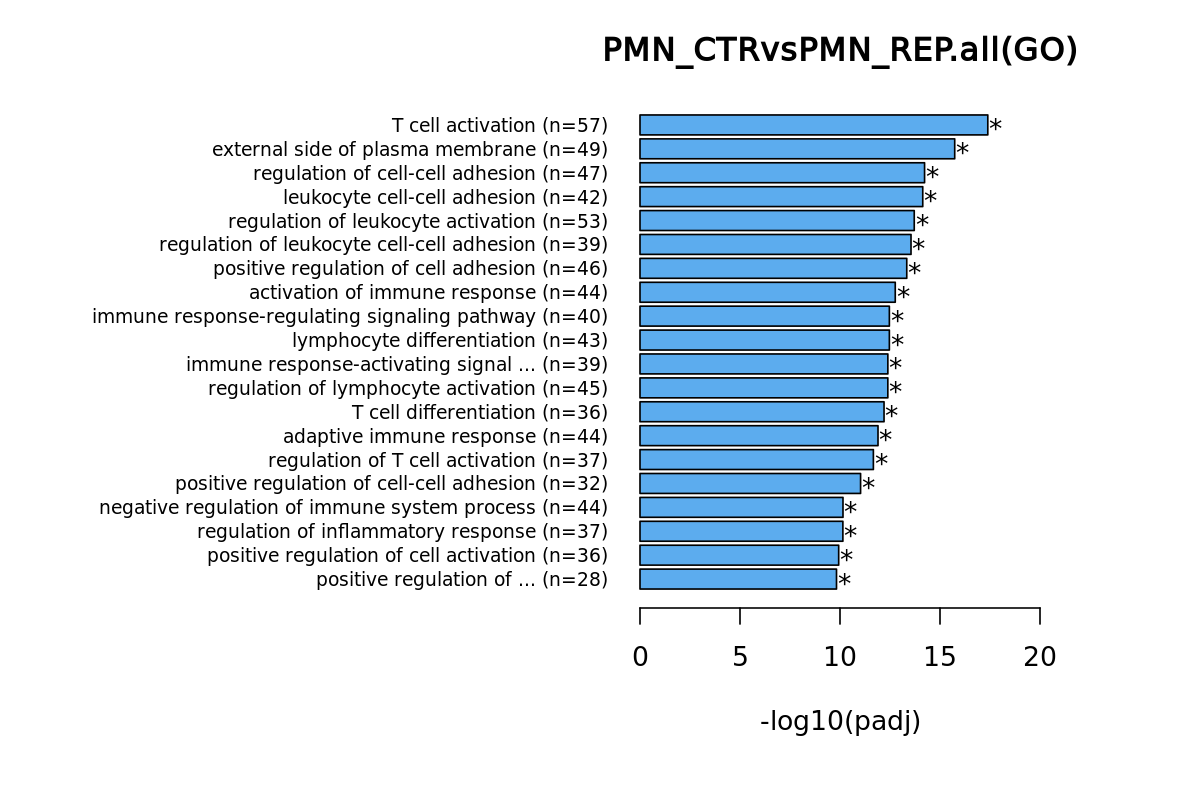


A

B

**GO Enrichment analysis of N1 DEGs**

**GO Enrichment analysis of N2 DEGs**

**Figure S3.** GO Enrichment Analysis. Top 20 significantly enriched terms in the GO enrichment analysis based on the DEGs of N1 and N2. The horizontal axis shows the enrichment score (-log10padj) and the vertical axis shows the terms with their respective number of differentially expressed genes.


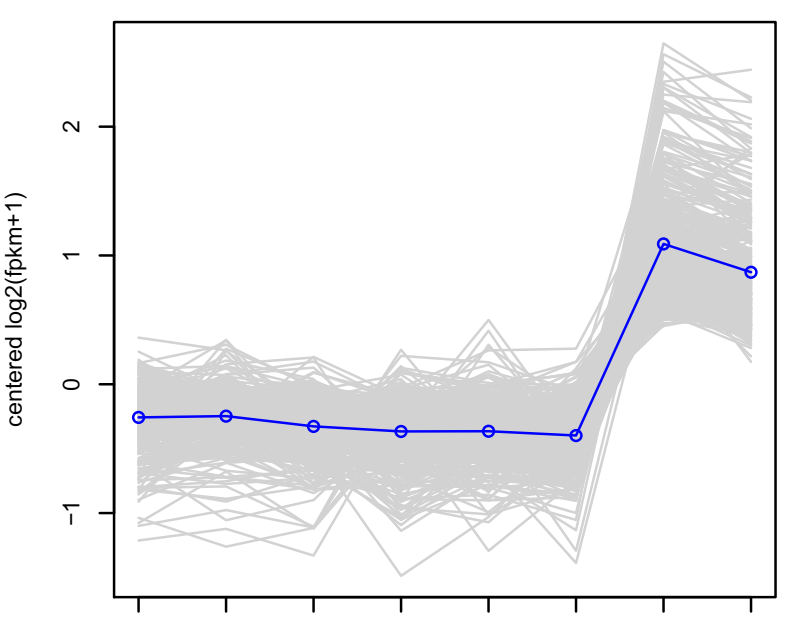


N_1

N_2

N_3

N2_1

N2_2

N2_3

N1_1

N1_2

**Figure S4.** Gene cluster of highly up-regulated genes in N1 compared with N and N2. Data is shown as centered expression (log2-transformed FPKM + 1). Each gray line represents the expression level of a particular gene, relative to the median expression level of all genes in that expression category. Blue lines represent the median expression level.


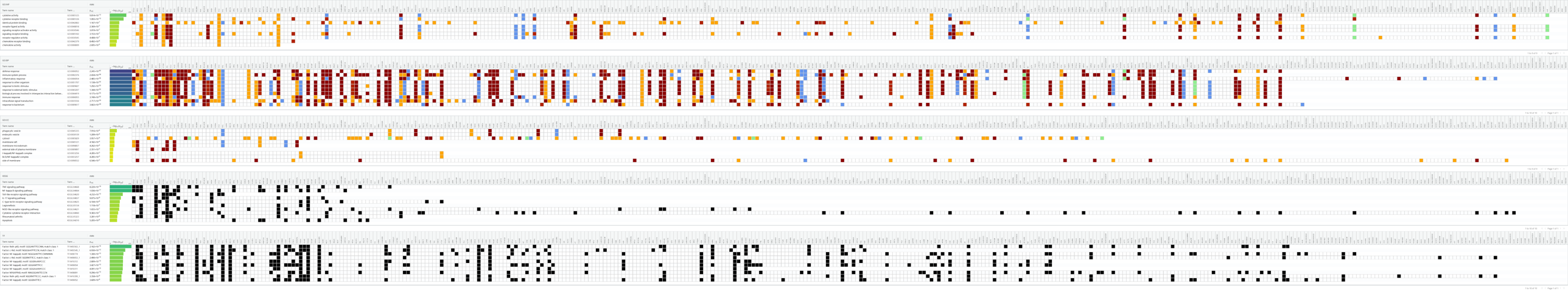

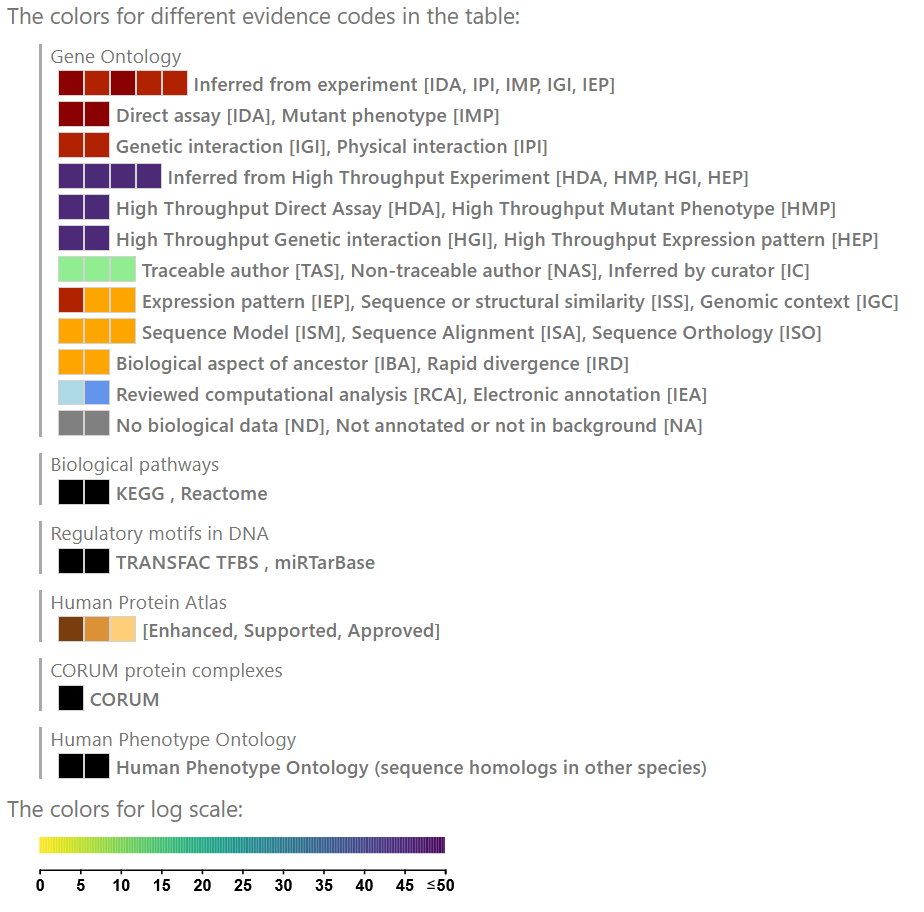

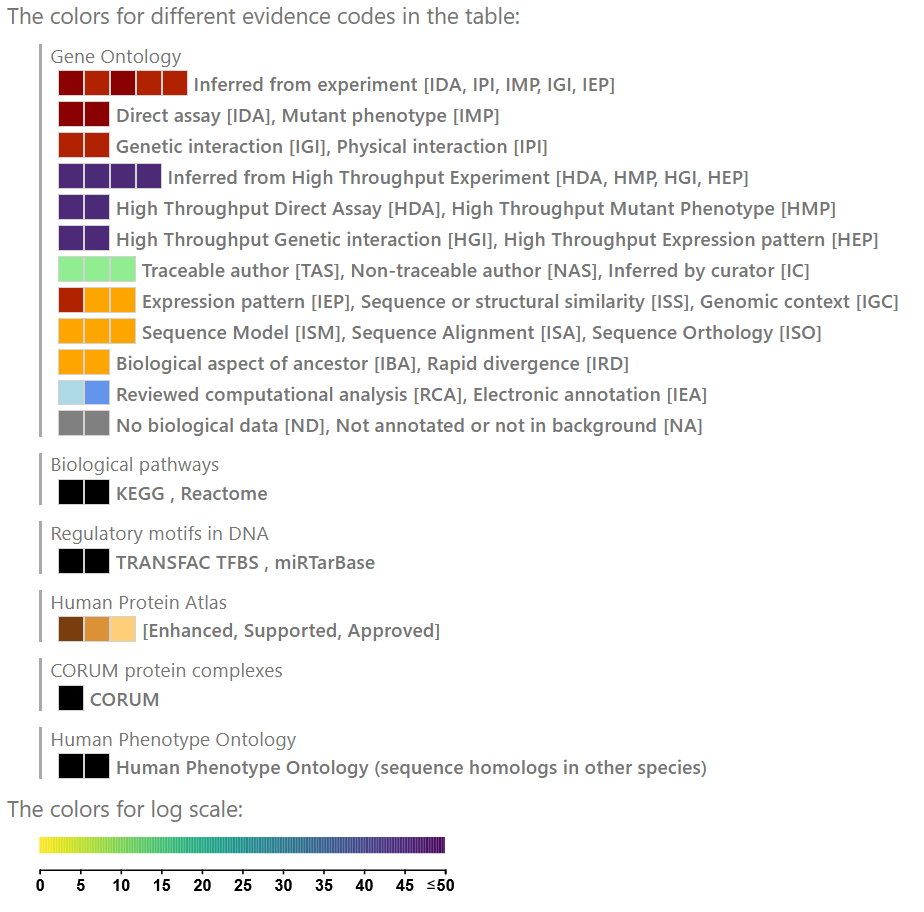


**Figure S5.** Functional enrichment analysis enrichment analysis by g:Profiler for GO, KEGG and TRANSFAC databases, the top 10 most enriched terms for each database.

**
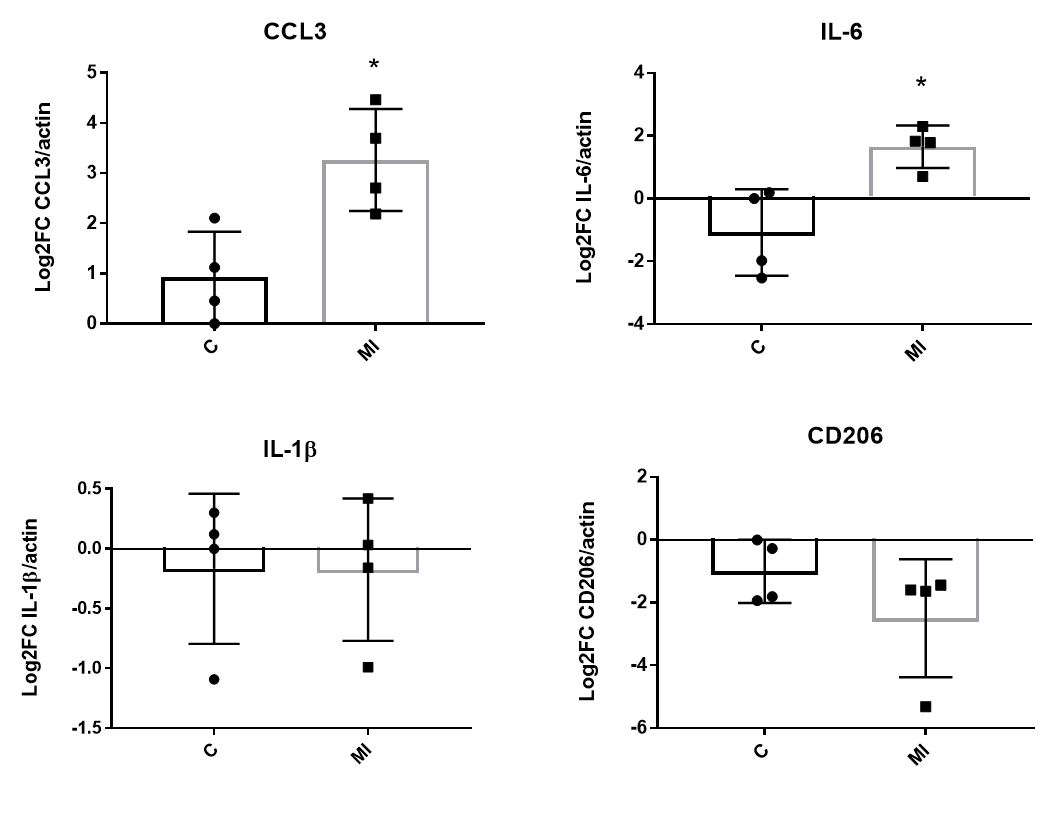
**

**Figure S6.** Gene expression of inflammatory/anti-inflammatory markers CCL3, IL-6, IL-1β, CD206 in neutrophils isolated from patients with myocardial infarction, in the first 24h post MI. *p<0.05. (C: control-healthy persons vs. MI: patients with MI).


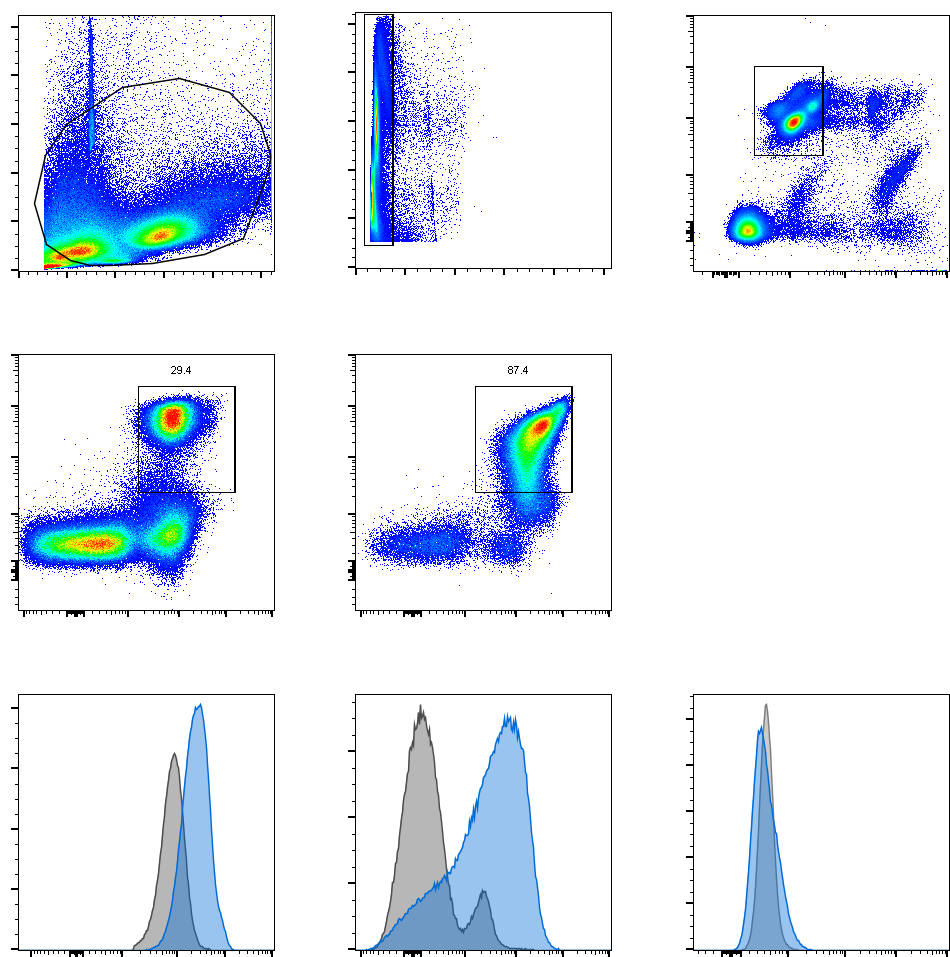


**A**

**B**

FSC-A

SSC-A

FSC-W

FSC-A

Live/Dead

CD45

CD11b

Ly-6G

CD11b

Ly-6G

CD11b

ICAM-1

CD206

**C**

**D**

**E**

**Naive**

**LPS**

Live leukocytes

Single cells

Neutrophils

Neutrophils


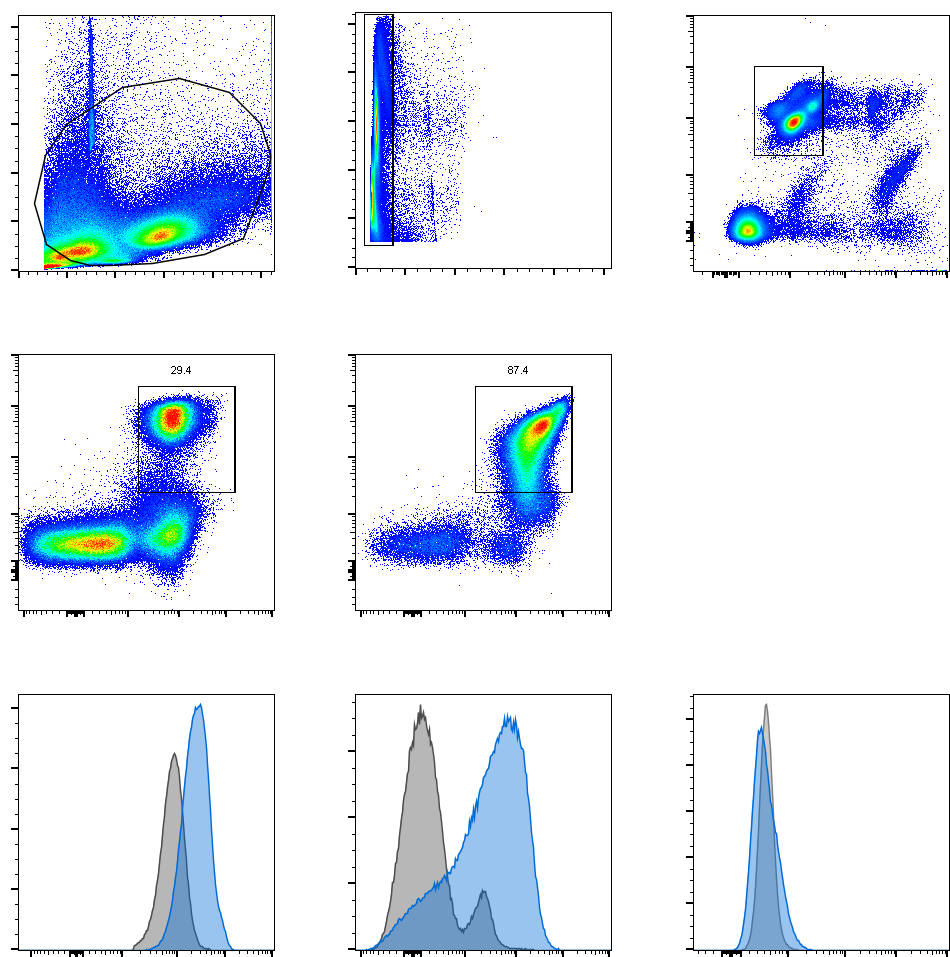


Counts

Fluorescence

Fluorescence

Fluorescence

Counts

Counts

**Figure S7.** Flow cytometry analysis of neutrophils in peripheral blood 24 hours after LPS stimulation: **(A)** Gating strategy. **(B)** Representative plots of blood neutrophils from naïve and LPS-stimulated mice, gated from live leukocytes. **(C)** Percentage Ly-6G^+^CD11b^+^ neutrophils out of live CD45^+^ leukocytes at 24h post-LPS stimulation. **(D)** Representative histograms and **(E)** quantification of mean fluorescence intensity (MFI) of CD11b, ICAM-1 and CD206 expression on blood neutrophils. Results are shown as mean ± SD (n=4-5). ****p<0.0001, ns: not significant.

**References:**

*(1). World Medical Association Declaration of Helsinki. Recommendations guiding physicians in biomedical research involving human subjects. Cardiovasc Res. 1997;35:2‐3.*
